# Exclusivity of Cultural Practices Within Emerging Disease Outbreak Responses in Developing Nations Leads to Detrimental Outcomes

**DOI:** 10.3389/fpubh.2021.686540

**Published:** 2021-07-06

**Authors:** Arnav Lal

**Affiliations:** School of Arts and Sciences, University of Pennsylvania, Philadelphia, PA, United States

**Keywords:** culture, emerging disease outbreak, Ebola (EBOV), Zika (ZIKV), traditional practices

## Abstract

A number of organizations provide aid and medical care to areas affected by emerging infectious disease outbreaks. This process oftentimes involves organizations traveling to developing areas and coordinating efforts on-site of the initial outbreak. Yet, the longevity and death toll of specific recent outbreaks and inability to effectively control them lead to unnecessary deaths and an unconstructive use of resources. While virtually all organizations justifiably point toward limited resources as an explanatory mechanism, this in itself does not excuse poor utilization of resources. Specifically, organizations systematically do not factor cultural practices into their disease responses. This is demonstrated in analyzing components of responses during 3 recent outbreaks occurring at different times and on different continents: Ebola in 2014 and 2019, and Zika in 2016. While systemic trends in these differential environments demonstrate the extent of the problem, fortunately, scientific innovations, collaboration with local individuals and leadership, and especially establishment of cross-cultural dialogue and response flexibility with the eventual development of effective behavioral change communication can help curb or mitigate this issue in the future.

## Introduction

In recent years, humans have increasingly spread further into undeveloped forest and wildlife habitats. Zoonotic or human territorial overlap increases the likelihood of pathogen crossover either directly into humans or via another organism. For example, the spread of commercial pig farms and housing into bat inhabited forests in Australia resulted in the first ever cases of Nipah Virus and Hendra Virus in Australia, Malaysia and other areas of Oceania (1). Such interaction is an inevitable process during the development of countries. As the sheer number and magnitude of emerging or re-emerging zoonotic diseases increase dramatically, the international community has responded to each major crisis through the deployment of aid, support, and medical staff. Their support is especially helpful (and oftentimes desperately needed) during times of rapid disease spread and when local hospitals lack the resources and staff to take on the health challenges posed to them. Organizations such as Médecins Sans Frontières (MSF), United Nations (UN), and World Health Organization (WHO) can often be located alongside the government and local hospitals at the location of outbreak. Concepts and real-life utilization of vaccines, ring immunizations, contact tracing, quarantine and personal protective equipment are just a few of the many techniques known and often used in outbreak responses. Yet somehow, despite increased knowledge, certain outbreaks still present difficulties which continue to challenge organizations.

Prior literature exists delineating problems and analyzing potential solutions for each independent major disease outbreak response. One such article focused on 2014 Ebola outbreaks in Sierra Leone (2) and thoroughly listed all forms of issues the Ebola response faced, including resource issues, fatigue and sociocultural challenges many which were limited due to costs and availability of resources. Therefore, an argument emerges that the deficiencies and lack of morale made it difficult for the response teams to interact with the community, inevitably creating cultural challenges such as mistrust and support toward traditional medicine. Of note, some cultural challenges were addressed (such as appointments of permanent teams to develop consistency) and the desire to develop community trust is listed as a goal to be attained in the future. However, most fundamentally, the significance of limited resources is stressed as the primary explanation for most challenges occurring in the outbreak response. Moreover, many leading officials also reported findings without directly mentioning means to improve cultural ignorance, rather chalking up challenges to a lack of resources (and accordingly the lack of resources is attributed to budget challenges). A US Congressional Research Service report delineated many of the challenges that the Ebola response team faced, including lack of services, workers, and (importantly) public support (3). The broad emphasis for resolving these constraints was placed on securing additional finances, not specifically on the use of available finances. Many other articles from both non-organizational research and the primary organization reports (WHO, MSF, UN) all argue with similar tone and structure in defending their policy (4–8). Through literature and press releases, the organizations argue that an increase in funding would lead to more efficacious outbreak responses. Despite the large volume of texts solely focused upon the financial factor during the disease outbreaks, few documents do include cultural differences in their analyses and points of action. For example, WHO published a document titled, “Culture matters: using a cultural contexts of health approach to enhance policy-making” (9). The overall message of the paper, namely that the WHO should “promote an understanding of the interrelationship between culture and health” demonstrates that in 2017, organizations recognized cultural differences as a significant issue which should at least be considered in the case of future outbreaks. Another notable contrast in dialogue is apparent in an article by Manguvo and Mafuvadze studying African traditional and religious practices and their effect on the efficacy of the Ebola response protocol (10). Despite the presence of these two texts, the majority of literature does not focus upon cultural impact upon response success.

It is challenging to compare outbreak responses globally due to the unique locational setting, nature of the outbreak, and influencing factors. This is magnified by the fact that each developing area of the world remains relatively isolated and thus developmental convergence is not (yet) observed. These unique conditions yield different cultures and traditions—which in part play a vital role in the magnitude and spread of human interaction within the area. Since human interaction is the primary way disease spreads, culture influences how quickly diseases spread. This can be demonstrated through analysis of different conflicts between cultural practices and epidemiological guidance within parts of the responses to three recent pathogenic outbreaks: Ebola (2014, 2019), and Zika (2016).

## Analysis

### Ebola Virus

Ebola is a filovirus that notably may spread to humans through the consumption of partially-cooked bat meat, and especially in the open-meat markets typically located in central and western Africa. Ebola is known to be the most virulent and easily spread after the person has passed away.

Certain actions undertaken during the outbreak response raise immediate concerns. First, response teams did not factor traditional medicine and practices performed by medicine-men of the specified areas. This concern has been underscored by case investigators as the most significant issue in the on-the-ground study by Boland et al. (2). While this issue may appear to be trivial, an estimated 70% of people in Africa seek advice from medicine-men (11). The worry from the allopathic medical community that traditional medicine may potentially spread the disease more than mitigating any dangers while potentially true, does not mean that traditional medicine can be ignored. In an entry by Carmem Pessoa da Silva, a WHO specialist, the information provided by the WHO and MSF to the public about staying safe did not include any reference or detail to traditional medicine (12). Yet, Pessoa also acknowledged that in communities without many resources, it was essential to prevent spread of infection through any means necessary—including even treating family members with a mere plastic bag as self-protection. While this statement proves that the WHO did suggest pragmatic (non-traditional/improvised) advice in the resource-limited settings, the absence of any information regarding the traditional medicine is evident, and this may likely have set the organization's efforts back, as at least some sick individuals would likely turn to the medicine-men they had known their entire lives. This claim does not discount the disparities and difficulties that would be associated with acknowledging the differences and procedures in how to understand the disease. Rather it instead is aimed at noting the importance of this potential avenue of support individuals may seek. In doing so, it is worthwhile to—at the very least—offer guidance toward the population with regards to conventional traditional medicine practices and their potential implications based upon what was understood about transmission, symptoms and convalescence. Doing so would not only present a potential deterrence to some while offering transparency in guidance to all.

The WHO and MSF both faced the broad challenge of societal distrust. According to MSF Project Coordinator John Johnson, people threw stones at MSF ambulances, which made it challenging for MSF to access certain neighborhoods. Those infected also refused to enter MSF hospitals, preferring traditional medicine and usually their own luck over the “westernized” hospitals, and this distrust grew wider as many patients didn't return home from the hospitals (13). Johnson claimed to never fully be able to control public resistance, even though any act of resistance could spoil the containment of the disease that the organization was trying to do. During the 2014 Ebola outbreak, a single unreported case by anyone in the population could potentially nullify the months of containment practices.

When Ebola remerged within the Democratic Republic of the Congo in 2019, the outbreak response team faced similar sociopolitical challenges different challenges to ones faced during the 2014–16 outbreak in Sierra Leone/Liberia. Specifically, in 2019, the cultural challenges that faced by conventional disease responses were dramatically escalated by sociopolitical influence upon situational driving factors. Per Human Rights Watch (14), the presence of bloody confrontations between militant factions were exacerbated by an election season, wherein some individuals may have even believed that the Ebola Virus was a political stunt (15)—highlighting the extent of systemic distrust likely designed by opportunistic groups within the location. Specifically, violence in Kivu (16), a region especially hard hit by Ebola and violence, was one of the most extreme challenges of addressing a disease outbreak in the midst of poor societal factors. Within this setting, a MSF Ebola center was attacked and another burned to the ground in North Kivu (17, 18). The reason behind the attack, while unclear, could likely be attributed to a number of factors, including a potential drive for violent factions to sow distrust within outbreak responses, a general distrust for the response practices or another reason. Less than a month after the attacks, MSF conceded that their Ebola response “failed to gain the upper hand” in the DRC (19). Despite the emerging conflicts and sociopolitical tensions, the primary reason cited in this statement was a lack of public support—although evidently all factors within the situation notably played a role within the overall course of the outbreak. Despite familiarity and the Ebola response and experimental vaccine, the operation still failed at least partly due to the public distrust. Without community trust, the response to the diseases were fully incapacitated with ~35% of new outbreaks lacking a known chain of transmission (19) demonstrating the implications of systematic fear and mistrusts of groups within the area. While this specific poor response is not fully attributable toward response teams (it rarely is), this case example substantiates the importance of sociocultural factors upon disease response success. While, in this specific case, the sociocultural challenges transitioned from internal to external as populational factions and social media^1^ became the predominant means of cultural challenges. During the new outbreak, strides were made to improve cultural clashes, including reflection by the Ebola Gbalo Research Group demonstrates an understanding of different settings, working with locals, and allowing locals to assist in directing the outbreak response as a result of the Sierra Leone Outbreak (20). This included recognizing families as critical to a patient's survival, and working with figures of authority within the locality. Despite attempts to reconcile societal issues from the 2014–2016 outbreak, surveyed individuals stated mistrust toward the disease response (72%) with 15% individual even expressing an intent to be non-compliant with regards to following directions/protocol of the response team (21). It has been theorized that unlike the 2014 Sierra Leone Outbreak, wherein teams composed of locals volunteered for activities such as restricting movement and contact tracing, the immediate inclusion of international responses was unable to develop agency within local systems, thus shifting the responsibilities toward outside entities—and thereby catalyzing potential distrust within the overall response (20). Hence again, the development and systemic presence of mistrust within society prevented an effective disease response.

Another major determinant for challenges for both the Ebola epidemics, but especially for the 2014 Sierra Leone outbreak was the response to traditional African burials. The Ebola virus infects others even when the human body has deceased, and so burial ceremonies, especially traditional ones, present a potentially dangerous inflection in which Ebola virus spreads to new human hosts. It has been estimated that every unsafe burial yielded 2.5 new cases (22), and this underscores the importance that burials play upon the effective containment of disease spread. The natural incidence and spread of natural fluids combined with their contact-based and potential aerosolization provide mechanistic substantiation to this worry. Thus, burial practices served as a hypothesized likely moment of probabilistic transmission, hence its importance to any disease containment epidemiology that would justifiably favor a method of burial that minimizes likelihood of spread, such as a cremation. However, local culture (including the influence of religion) not only frowned upon cremation but also believed in cleansing the body for burial. This process involves cleaning the body of any food they have eaten, oftentimes using bare hands. The obvious contact with body fluids creates a striking clash between the orders of non-governmental organizations and the structural mandates of culture. As a pathway to a solution, the World Health Organization states, “providing the opportunity for a safe yet dignified burial is an opportunity to respect cultures and traditions and provides comfort to those left behind” (23). While this statement may appear to appease the problems at hand, further analysis of the implications demonstrates the natural challenges that arise when conflicts exist between health safety and cultural practices. The subsequently recommended WHO Ebola burial procedures did not list the Ebola virus burial procedures with those of the Marburg virus (which affected municipalities of Germany, with totally different culture and burial practices), but despite the presence of guidance with respect to religious observance, the guidance offered little more to accommodate the conventional West African cultures and traditions (24). Moreover, the document depicts individuals (with PPE off) asking locals regarding their burial preferences, an unrealistic and unimaginably challenging concept to carry out even without large magnitude of infections but especially challenging for individuals overworked and overburdened with their dangerous job. Guidance listed within this document, while potentially effective at developing a compromising position between health and culture does so with ideas that are non-implementable in a pragmatic sense (I was unable to find any instance of WHO-advised burial strategies being implemented in on-the-ground reports). While it is fair that procedural focus is upon safety over culture and tradition, the notion that pragmatic suggestions are necessarily coupled with ones that would be detrimental to public health provides a false solution. The inevitable retraction of all cultural elements given the lack of a pragmatically viable compromise was therefore understood to be a betrayal for the family members who believed in afterlife and believed in careful treatment of the dead. Moreover, field documents report that the Ebola “Dead Body Management Teams” not only did not communicate with the families (a violation of the unrealistically delineated WHO protocol), but also took the individuals who were (at the time) alive but on death's doorstep with them as well in an effort to curb the spread of the disease while saving another trip back, an action whose implications were likely exacerbated by a lack of communication (25). Therefore, it is inevitable that some people never reported being sick or having deceased family members to prevent family members from being buried without consent—these actions obviously undermined much of the Ebola disease prevention contact tracing that the organization worked toward. Moreover, when health workers entered the home of a potentially infected individual, they wore a bulky bright Personal Protective Equipment (PPE)—for personal safety purposes. To the community, the individuals looked like space aliens, especially with their mouths completely covered up, which prevented them from communicating properly (26). This effectively curtailed the already limited communication, which bred even more distrust. While the necessary emphasis upon safety especially during burials is fully justified, an approach that offers capacity for inclusion of cultural elements only with sacrifice of the procedural safety of individuals will lead to non-compliance. The recognition of cultural factors within burial practices makes evident the extent to the challenges and while compromise was likely the most optimal solution if done correctly, its application in this case is simply non-implementable during an epidemic. Thus, the true challenge occurs in developing procedures that adhere to this compromise without susceptibility of non-practice within a real setting.

Finally, as is normal in every other infectious disease containment program, patients suspected of carrying Ebola virus were subjected to quarantine (27). However, developing nations are communal and oftentimes lack the infrastructure (phones, etc.,) to maintain relationships while quarantined, and the isolation wards shown in a documentary of an MSF treatment facility displayed a 4-wall tarped and confined room with only one small blurry (translucent) window (28). This, however, was not the only type of quarantine shown in the video, with other more open yet safe options. The fact that there are other options that use less building material and space, are equally safe, and allow communication to continue signifies the failure of MSF to understand the community in which they were offering assistance. Obviously, such challenges concern financial issues—yet it is important to recognize that the use of given funds to more socially acceptable options would likely yield better outcomes. Simple changes like communication, more adequate quarantine zones, and change of PPE apparel would dramatically lower sociocultural impacts of Ebola responses.

### Zika Virus

Zika is carried primarily by the mosquitoes *Aedes aegypti*. In 2015, a Zika epidemic occurred in Brazil with cases reported across South and Central America). Zika infections display flu-like symptoms and the transmission of Zika to a pregnant woman may yield conditions of microcephaly for the baby (a condition in infants marked by abnormal head growth and mental deficiencies). Zika is also spread through sexual transmission and blood transfusions, though the primary means remains mosquito bites (29). Historically, Zika virus was predominantly identified in Central Africa and Southeastern Asia in the late 1900's (30), and hence its presence within South American countries was not anticipated. The incidence of mild symptoms (e.g., a skin rash) associated with the illness and no reported deaths in early 2015 was not as worrisome as the Zika epidemic spread would eventually become. This change was mediated by the observance of microcephaly and other neurological disorders within the population such that within 1 year, the epidemic became a public health emergency (30).

Given the necessity of still water for the replication of *Aedes aegypti*, a main tactic in stopping the spread of Zika involved eliminating accessible pools of water or using insecticide, therefore depriving mosquitoes of water bodies to lay eggs and reproduce in. However, Brazilians in favelas and many other areas of the country were accustomed to preserving their water in tanks especially during the summer, and dumping this resource to prevent the spread of mosquitoes made little pragmatic sense to them, given that their richer neighbors didn't have to dump their water (they had ample funds to secure the water storage, or use insecticide) (31). From an epidemiological perspective, the higher incidence of infection, caused by the greater ability of mosquitoes to infect and reside within the open favelas implied that the presence of still water within the location of the favelas likely had a higher probability of being implicated within the overall disease response. Nevertheless, given a disproportionate response problem exacerbated by the income-gap of residents that looked like targeting based upon income, few individuals elected to dump or cover their water, electing instead to ignore the orders of the Brazilian national health department and police.

Brazilian Traditional Medicine is a factor to the epidemiological history of the region, with its unique influences traceable back to the different cultural groups that inhabited the country in the past (32). Remarkably, this traditional medicine has developed intimate knowledge of plants and other entities within the environment. Brazilian traditional medicine has grown to the point that many people prefer to go to the medicine-man of the village over allopathic care (33). When Zika patients visit local medicine-men, they may often be mis-diagnosed as malaria patients since both Zika and Malaria share common symptoms. To make matters worse, Brazilian traditional medicine also includes the use of ground mosquito parts and other insects in their healing methods (34). Any use of this treatment type may result in more people contracting Zika. While the influences of traditional medicine are not as well-documented as in the Ebola Virus outbreaks and traditional medicine is not thought to play a major role in transmission, the presence and influence of traditional medicine within the area likely played some role in the overall outbreak transmission.

Finally, one method (from the UN General Council and WHO) for preventing microcephaly in Brazil involved maximizing availability of contraceptives and abortion care and conducting social science research on abortion in nations afflicted with Zika (35, 36). Since the most concerning implications of Zika Virus infections target children, the prevention of pregnancy and/or birth is an obvious means of preventing long-term implications of the infection. The prevention of pregnancy upon developing knowledge of its implications is a cultural topic worth exploring, yet it is challenging to dissect the different roles that are played by independent factors in determining the outcomes of guidance. Whether it was governmental factors, outside organizations, community-based determination, or a combination of factors is difficult to determine with the lack of on-the-ground evidence. Notably, however, anonymous public health officials have offered some insights into other factors which influence Zika transmission with respect to pregnancy. Phenomenological studies post-infection described how despite the guidance offered by healthcare professionals for the women's partners to use condoms during pregnancy, they anticipated that the compliance for this procedure was virtually non-existent due to the severe unpopularity of the practice (37). This, exacerbated by the false notion that individuals believe the baby within pregnant mothers had “already formed” (37) and hence further transmission would not affect baby growth, led to the possibility that transmission to pregnant mothers via sexual intercourse remained a viable route of infection. Hence, it is possible that fear may have been a strong driving force toward decision making with regards to pregnancy, given that health official advice—when states against cultural norms—quite evidently did not yield change in action.

This leads to the final means of symptom prevention: abortion. Brazil was and is a predominantly Catholic nation, and the Catholic Church openly opposes all forms of abortion. Not only did many women not desire abortions, but even if they did, Brazilian women reported social stigma if they chose to perform one, according to Human Rights Watch (38). The funding of research on abortion clinics and construction of abortion clinics in 2014 therefore appears to be an evident misutilization of capital, and would present an obvious symbol of the conflict between culture and public health. Even increased access to contraceptives would likely be met with limited success due to the fact that many women in these nations “cannot exercise the control…. under what circumstances become pregnant,” according to UN human rights Chief Zeid Ra'ad al-Hussein (36). Moreover, such a construction project or contraceptive drive is a prime example of when even extra finances would not help an international aid effort that ignores cultures and practices. Adding more abortion clinics would simply spur more division, moreover would also be a legal challenge as abortions are illegal in many South American nations (39). Notably, the UN also invested time and resources to treat the babies with microcephaly, and they provided comprehensive information pertaining to child development, breastfeeding and nutrition (40). However, the resources and analysis dedicated in any form to abortion facilities and staff appear to be efforts that could have been better spent on general healthcare for the Brazilian populous during an outbreak.

## Discussion

In light of the case studies presented, it is clear that cultural and social problems exist in infectious disease responses, and their importance to the overall success of the project cannot be ignored due to the fact that cultural interactions determine disease spread. The major challenges detected within this manuscript is the fundamental conflict between health/safety and sociocultural practice. Examples of this span all three infectious diseases described within the paper, from burial practices with regards to Ebola, to sexual transmission during pregnancy for Zika Virus, to quarantine methodologies for Ebola. Each of the three examples have an epidemiological “solution” in the form of complete preventative burials, condom use, and different materials/layouts for quarantine, respectively, yet the public action within all three are starkly different. Whereas the different quarantine structures form a solution that is able to maintain health and safety while also allowing the continuation of cultural activities, the Ebola burials demonstrate an activity conducted with an unwilling populous, met with resistance and unwillingness of the population. In comparison, condom use during the Zika outbreak represented a case of individual mediated action, which likely was not conducted to any extent: thus, allowing for the cultural practices of the region while maximizing risk of dangerous transmission. Hence, there exists different sociocultural extents and natures to problems which affect organizations, ranging from those unavoidable to ones that can be resolved.

This variability reveals the scope of approaches that can be utilized in order to prevent the occurrences of major issues of this nature. Evidenced by physical quarantine structure during the Ebola Virus epidemic, wherein the creation of a different style of quarantine system would be more amenable to the cultural practices of the area, if there exists a means of fully isolating scientific issues from epidemiological ones by means of solution creation, this forms the most parsimonious solution to the challenges faced.

Another available means of resolution during outbreaks involved enlisting local help and guidance. While it may be the case that certain procedures and protocols may feel alien, the recruitment and collaboration with entities, people and established organizations offer a means of assisting the assimilation of epidemiological practices within the communities. While the existing infrastructure within developing nations may not be able to handle the epidemic, much of the populous would be likely to support and assist in various necessary activities of the disease response, and their further integration into the overall disease response may likely allow more of the population to be more sympathetic toward the overall disease response. An example of this is delineated by the Ebola Gbalo research (20), wherein the further incorporation of locals appears to be associated with further trust of the epidemiological approach, as exemplified by the increase of trust during the 2014 Sierra Leone outbreak vs. the 2019 DRC outbreak due in part to the higher prevalence of locals (including volunteers) serving roles including contact tracing, care, and blocking travel in highly infected areas.

Local leadership offers another critical element that can determine the success of an outbreak response. For example, both Christian and Islamic leaders during the Ebola outbreak would likely be able to draw upon their religious organization to advocate for pauses in people touching the dead during the outbreak. This may have greater significance to certain individuals over the guidance or signs from foreign organizations. The use of on-site institutions such as churches and local leaders is a cost-effective and efficacious method which every organization should adopt.

Other organizations may also be critical to the overall success of the outbreak response. For example, cultural anthropologists offered to help improve the practices of the WHO and MSF during the Ebola outbreak (41). Though their offer of help was declined [but accepted during the 2019 outbreak (20)], such offers can prove to be invaluable for any organization. Such support should be considered especially during the *planning* phase of an outbreak response, while organizations have time to predict and prevent such cultural conflicts, and this is especially easy to perform in cases of a recurrent disease in a similar geographic outbreak. This approach will require the responses to be more flexible.

Finally, there inevitably will exist many problems which are simply so dangerous pathologically (for example, Ebola burial practices) that they necessitate obligatory action. With regards to these issues, which by their nature are the most important due to the magnitude of their potential effect, the most likely solution involves long-term elimination of risk factors to public health. Doing so implies the incorporation of community-centered health education globally. By helping individuals learn the prerequisites and techniques necessary to maintain their health—and for them to subsequently empirically observe the impacts of the change—is likely the only way to eliminate cultural challenges in the future. The introduction of community-centered health education will almost likely lead to behavioral change communication (and even potentially cultural change) that can eliminate major competition between cultural factors and epidemiology. By developing changes in behavior that align with public health policies, many of the challenges associated with infectious diseases responses will be eliminated and it is also likely that the volume of responses may also decline.

## Conclusions

Culture plays an extremely large role in infectious disease outbreaks, as it determines which ways a disease will spread throughout a community, and is variable throughout the developing nations. Organizations need to be aware of the cultural practices and religious beliefs of certain areas, especially before even entering an outbreak area. Optimistically, the importance of culture and human-centered outbreak responses have grown. If the organizations are able to adopt a more human-centered approach, their staff will have to work for a shorter amount of time, require less resources and will be able to save more human lives.

## Data Availability Statement

The original contributions presented in the study are included in the article/supplementary material, further inquiries can be directed to the corresponding author/s.

## Author Contributions

The author confirms being the sole contributor of this work and has approved it for publication.

## Conflict of Interest

The author declares that the research was conducted in the absence of any commercial or financial relationships that could be construed as a potential conflict of interest.

## Footnotes

^1^https://www.cfr.org/blog/disinformation-and-disease-social-media-and-ebola-epidemic-democratic-republic-congo
